# Handgrip strength is independently associated with physical quality of life in patients undergoing maintenance hemodialysis: a cross-sectional study

**DOI:** 10.3389/fnut.2024.1478209

**Published:** 2024-12-03

**Authors:** Chunlei Li, Xiangyou Pan, Shilin Xu, Jianguang Hu, Xiaoshi Zhong, Luona Wen, Jingxian Qiu, Rongshao Tan

**Affiliations:** ^1^Department of Clinical Nutrition, Guangzhou Red Cross Hospital of Jinan University, Guangzhou, China; ^2^Guangzhou Institute of Disease-Oriented Nutritional Research, Guangzhou Red Cross Hospital of Jinan University, Guangzhou, China; ^3^Department of Nephrology, Guangzhou Red Cross Hospital of Jinan University, Guangzhou, China

**Keywords:** muscle strength, quality of life, physical health, sarcopenia, maintenance hemodialysis

## Abstract

**Objective:**

We aimed to identify the association between Health-related quality of life (HRQoL) and muscle strength in patients undergoing maintenance hemodialysis (MHD).

**Methods:**

In this cross-sectional study from March 2021 to December 2021, 110 MHD patients with a mean age of 63.9 ± 13.0 years and a median dialysis vintage of 25.5 (12.0–52.3) months, were enrolled at a hemodialysis center in Guangzhou city, China. HRQoL was assessed using the Short Form 36 Health Survey (SF-36) and converted into the Physical Component Summary (PCS) and the Mental Component Summary (MCS). The groups were assigned according to the mean score of PCS and MCS, and those with higher PCS/MCS scores (high-PCS/MCS) were compared with those with lower PCS/MCS scores (low-PCS/MCS). Independent factors were evaluated using multivariate analysis. Muscle strength was estimated by handgrip strength (HGS).

**Results:**

The mean HGS was 23.7 ± 9.60 kg in men and 14.3 ± 5.30 kg in women. Compared to the high-PCS group, the low-PCS group had older age, higher levels of creatinine, total cholesterol, high-sensitivity C-reactive protein (hsCRP) and interleukin-6 (IL-6), and had lower HGS (all *p* < 0.05). After adjusting for confounding factors in different models, the five-model multivariate binary logistic regression analyses revealed that HGS was the only independent factor in PCS domain, but not in MCS.

**Conclusion:**

HGS may be an independent factor of poor HRQoL in MHD patients, particularly in relation to physical health. The management of muscle strength may improve the HRQoL in MHD patients.

**Clinical trial registration:**

The study was registered at https://www.chictr.org.cn/ as ChiCTR2100053790.

## Introduction

Maintenance hemodialysis (MHD) serves as a life-sustaining treatment for individuals with end-stage renal disease (ESRD). Although the survival rate of ESRD has improved over time, patients undergoing MHD have a lower quality of life compared to the general population (1). Health-related quality of life (HRQoL) reflects the impact of disease and healthcare on patients’ functional status and their subjective perception of life (2), and it is affected by factors such as psychological health, physical state, level of independence, and social relations (3). In MHD patients, studies have shown that HRQoL is a strong and independent predictor of mortality and hospitalization (4–8). Evaluating and analyzing HRQoL in MHD patients is an important objective. Various generic and disease-specific questionnaires have been utilized to evaluate the quality of life in patients with renal disease (9). The Medical Outcomes Study Short Form 36 Health Survey (SF-36) is a widely recognized generic instrument for assessing HRQoL in ESRD patients (2, 10). There are differences in the HRQoL of hemodialysis patients across different countries and regions (11).

As renal function deteriorates, MHD patients experience physical inactivity, malnutrition and muscle wasting, which together make them more prone to sarcopenia (12). Sarcopenia, characterized by a combination of low muscle mass, strength and function, (13) is a condition related to aging with adverse clinical outcomes, including increased mortality rates, diminished mobility, and an elevated risk of falls and fractures (14, 15). Previous studies on older individuals have suggested that muscle strength, rather than muscle mass, influences factors such as quality of life, mortality risk and physical performance (16–18). While sarcopenia has been linked to reduced HRQoL in community-dwelling older adults (19, 20), the impact of sarcopenia and its components, such as muscle strength and muscle mass, on HRQoL in patients undergoing MHD remains underexplored (21–23).

The objective of this cross-sectional study was to examine the relationship between HRQoL and sarcopenic parameters, with a specific focus on muscle strength, in Chinese patients undergoing MHD.

## Methods

### Study design and participants

A single-center cross-sectional study was conducted. Patients were recruited from March 2021 to December 2021 in the hemodialysis center of our hospital. This study was conducted in accordance with the principles of the Declaration of Helsinki and was approved by the Ethics Committee of Guangzhou Red Cross Hospital, Jinan University (No.2021–066-01). The study was registered on the Chinese Clinical Trial Registry (ChiCTR2100053790).^1^ Informed consent was obtained from each participant before enrollment.

The inclusion criteria were as follows: (a) dialysis vintage more than 3 months, three times a week, 4 h each session, (b) age 18 years or older, (c) informed consent. The exclusion criteria were: (a) patients with pacemakers, (b) a history of severe infections, trauma or surgery within the past 3 months, (c) patients with diabetic feet, or amputations, (d) those with physical disabilities or who could not cooperate with the study for other reasons, (e) patients with malignant tumors.

### Health-related quality of life

HRQoL was assessed using the 36-item Short-Form Health Survey (SF-36) (24). This tool is a patient-related questionnaire that generates scores across eight subscales: physical functioning (PF), role limitations due to physical problems (RP), bodily pain (BP), general health (GH), vitality (VT), social functioning (SF), role limitations due to emotional problems (RE), mental health (MH). Each scale is scored from 0 to 100. Two health component summary measures, the Physical Component Summary (PCS) and Mental Component Summary (MCS), were calculated from eight domain subscales for easier and simpler interpretation and analysis (25, 26). A higher score on these summaries indicates better HRQoL. The SF-36 scores use norm-based scoring in which the scale and component summary scores have a mean of 50 and standard deviation of 10 in the US general population. For better international comparability, patients in this study were allocated into two groups based on the mean of 50, which originated from the U.S. population. In each summary component, scores ≥50 were designated as the high-PCS or high-MCS (better HRQoL) group, and the scores <50 was designated as the low-PCS or low MCS (poorer HRQoL) group.

### Handgrip strength

Handgrip strength (HGS) was measured using the BASELINE digital Grip Force Tester (12–0091, Fabrication Enterprises Inc., United States). Measurements were performed on the non-fistula hand before the hemodialysis session. Participants were seated in a straight-backed chair with their feet flat on the floor, their shoulders adducted, and their elbow flexed at 90°. The forearm was kept in a neutral position. The arm should not be supported by the patient, examiner, or by an armrest. The dynamometer was held vertically in line with the forearm. The hand was positioned with the thumb around one side of the handle and the four fingers around the other side. For each patient, measurements were repeated three times and the highest score was used in our analysis. The interval between the three measurements was at least 1 min. Grip strength was read in kilograms (kg) from the outside dial and recorded to the nearest 1 kg on the data entry form. According to the consensus of the Asian Working Group for Sarcopenia (AWGS) (13), low hand grip strength (low-HGS) was defined as HGS <28 kg for men and < 18 kg for women.

### Demographic, clinical and laboratory parameters

Demographic (age, gender), clinical data (dialysis duration, history of diabetes, primary disease) were collected by retrieving medical records and asking the patient directly. Blood samples were collected before dialysis sessions for serum dosages of blood urea nitrogen (BUN), creatinine, phosphorus, hemoglobin, albumin, prealbumin, total cholesterol (TC), triglyceride, high-sensitivity C-reactive protein (hsCRP), interleukin-6 (IL-6), interleukin-1*β* (IL-1*β*), tumor necrosis factor-*α* (TNF-*α*), transforming growth factor-*β* (TGF-*β*). The Kt/V was calculated according to Daugirda’s formula (27).

### Anthropometric measurement

Dry body weight and height were measured to the nearest 0.01 kg/0.1 cm, and body mass index (BMI) was calculated as dry body weight divided by squared height (kg/m^2^). We also assessed triceps skinfold thick ness (TSF) and mid-upper arm circumference (MAC) after the hemodialysis sessions with the skinfold caliper (Guangdong Xiangshanweihua Corporation Ltd., EH101, China) and a flexible plastic, non-stretchable tape. Mid-arm muscle circumference (MAMC) was calculated using the following equation: (28).

$\mathrm{MAMC}\;\left(\mathrm{cm}\right)=MAC\;\left(\mathrm{cm}\right)-\left[TSF\;\left(\mathrm{cm}\right)\times \pi \right]\text{.}$

### Body composition measurement

Whole-body composition was measured using a body composition analyzer (Multiscan 5,000; Bodystat, Isle of Man, United Kingdom) by bioimpedance spectroscopy analysis (BIS). Body fat mass and lean body mass were measured. The equations for calculating appendicular skeletal muscle (ASM) and appendicular skeletal muscle index (ASMI) are according to Adrian’s formula (29).

### Sarcopenia criteria

Sarcopenia was diagnosed as low HGS and low ASMI on the basis of the criteria proposed by AWGS (2019): (13) (1) ASMI <7.0 kg/m^2^ for men and < 5.7 kg/m^2^ for women; (2) HGS <28 kg for men and < 18 kg for women.

### Statistical analysis

Statistical analyses were performed using SPSS version 22.0 statistical software (IBM Corp., Armonk, NY). All variables were tested for normality using the Shapiro–Wilk normality test. Continuous variables with a normal distribution were presented as the mean ± standard deviation, and those with non-normal distribution were described as median (25–75% interquartile range). Categorical variables were presented using percentages. Participants were divided into high-PCS/MCS and low-PCS/MCS groups. Differences between the 2 groups were assessed using a student’s t-test, Mann–Whitney U test, or chi-squared test, as appropriate. Normally distributed variables were compared using the t-test. For variables that did not exhibit a normal distribution, the Mann–Whitney U test was utilized. Associations between categorical variables were estimated using the chi-squared test. The two-tailed Fisher exact test was used for dichotomous variables. Variables with *p*-values <0.05 on comparison between the groups were subsequently analyzed using multivariate logistic regression to identify the independent factors of low-PCS/MCS by using a forward-stepwise method. A two-tailed *p*-value <0.05 was considered to indicate statistical significance.

## Results

### Comparison of clinical characteristics between the high- and low-PCS/MCS groups

Comparison of clinical characteristics between high-and low-PCS/MCS groups are shown in Tables 1, 2. The number of participants in the high-PCS group was smaller than that in the low-PCS group. Compared to the high-PCS group, the low-PCS group had older age, higher levels of total cholesterol, high-sensitivity C-reactive protein (hsCRP) and interleukin-6 (IL-6), as well as lower levels of creatinine and reduced hand grip strength (all *p* < 0.05; Table 1). There were no differences in gender, BMI, dialysis vintage, BUN, kt/V, hemoglobin, albumin and prealbumin between the high-and low-PCS groups (Table 1). Only total cholesterol levels showed significant differences between the low-MCS and high-MCS groups. Compared to the high-MCS group, the low-MCS group had lower levels of total cholesterol (*p* = 0.034; Table 2).

**Table 1:** Comparison of clinical characteristics of the high-and low-PCS groups.

Variables	High-PCS^†^ *n* = 36	Low-PCS^†^ *n* = 74	*p* ^‡^
Age (years)	58.5 ± 12.4	66.5 ± 12.5	0.002
Women [n (%)]	12 (33.3)	28 (37.8)	0.645
BMI (kg/m^2^)	22.2 ± 2.70	23.4 ± 4.00	0.124
Dialysis vintage (months)	35.0 (11.5–67.5)	24.0 (12.0–50.3)	0.456
Cause of end-stage renal disease [n (%)]			0.504
Diabetic nephropathy	11 (30.6)	28 (37.8)	
Hypertensive nephropathy	7 (19.4)	7 (9.50)	
Polycystic kidney disease	2 (5.60)	2 (2.70)	
Unknown causes	12 (33.3)	30 (40.5)	
Other^§^	4 (11.1)	7 (9.50)	
Diabetes mellitus [n (%)]	14 (38.9)	42 (56.8)	0.079
BUN (mmol/L)	29.3 (25.0–32.7)	26.7 (22.1–32.0)	0.055
Creatinine (μmol/L)	1,075 ± 313	948 ± 278	0.034
Kt/V	1.40 (1.20–1.50)	1.30 (1.20–1.60)	0.679
Serum phosphorus (mmol/L)	2.10 (1.70–2.60)	2.20 (1.80–2.60)	0.821
Hemoglobin (g/L)	95.3 ± 17.4	97.7 ± 16.6	0.470
Albumin (g/L)	38.0 ± 2.90	36.7 ± 3.30	0.065
Prealbumin (mg/L)	347 ± 60.7	321 ± 69.7	0.058
Triglyceride (mmol/L)	1.70 ± 1.00	2.20 ± 1.80	0.113
Total cholesterol (mmol/L)	4.10 ± 1.00	4.60 ± 1.10	0.021
hsCRP (mg/L)	2.10 (1.00–7.60)	4.30 (1.70–8.70)	0.024
IL-6 (pg/mL)	6.80 (4.60–10.7)	9.40 (6.30–16.5)	0.026
IL-1*β* (pg/mL)	5.00 (5.00–7.20)	5.00 (5.00–7.50)	0.962
TNF-*α* (pg/mL)	12.8 (11.7–17.0)	14.3 (12.5–16.5)	0.469
TGF-*β* (pg/mL)	179 (1.00–680)	172 (42.2–460)	0.872
HGS (kg)	24.8 ± 9.80	17.9 ± 8.40	<0.001
Low-HGS [n (%)]	22 (61.1)	66 (89.2)	0.001
MAMC (cm)	21.3 ± 3.00	21.9 ± 3.50	0.383
Lean mass (kg)	46.4 ± 9.40	45.6 ± 10.4	0.684
Fat mass (kg)	13.4 ± 4.80	16.8 ± 6.60	0.008
ASMI (kg/m^2^)	6.80 ± 1.00	6.80 ± 1.20	0.752
Sarcopenia [n (%)]	13 (36.1)	26 (35.1)	0.920

PCS, Physical Component Summary; BMI, body mass index; BUN, blood urea nitrogen; Kt/V, dialysis efficiency index; hsCRP, high-sensitivity C-reactive protein; IL-6, interleukin-6; IL-1β, interleukin-1β; TNF-α, tumor necrosis factor-α; TGF-β, transforming growth factor-β; HGS, hand grip strength; MAMC, mid-arm muscle circumference; ASMI, appendicular skeletal muscle index. Continuous variables are expressed as mean ± standard deviation or median with interquartile range in case of nonnormally distributed data, and categorical variables are expressed as percentages.

† Patients were divided into the 2 groups by whether PCS ≥ 50.

‡ Obtained from two-sample t-test, Mann–Whitney U test, or chi-square test as appropriate.

§ Other: IgA nephropathy, membranous nephropathy, drug-induced nephropathy.

**Table 2:** Comparison of clinical characteristics of the high- and low-MCS groups.

Variables	High-MCS^†^ *n* = 94	Low-MCS^†^ *n* = 16	*p* ^‡^
Age (years)	64.2 ± 13.2	62.1 ± 12.1	0.546
Women [n (%)]	35 (37.2)	5 (31.2)	0.646
BMI, (kg/m^2^)	23.2 ± 3.80	21.7 ± 2.60	0.142
Dialysis vintage (months)	24.5 (11.0–51.3)	41.0 (16.0–72.8)	0.214
Cause of end-stage renal disease [n (%)]			0.680
Diabetic nephropathy	35 (37.2)	4 (25.0)	
Hypertensive nephropathy	12 (12.8)	2 (12.4)	
Polycystic kidney disease	3 (3.20)	1 (6.30)	
Unknown causes	34 (36.2)	8 (50.0)	
Other^§^	10 (10.6)	1 (6.30)	
Diabetes mellitus [n (%)]	49 (52.1)	7 (43.8)	0.535
BUN (mmol/L)	27.3 (23.6–32.3)	29.0 (23.7–31.8)	0.845
Creatinine (μmol/L)	985 ± 313	1,015 ± 152	0.559
Kt/V	1.30 (1.20–1.50)	1.30 (1.20–1.60)	0.665
Serum phosphorus (mmol/L)	2.10 (1.80–2.50)	2.50 (1.90–2.90)	0.168
Hemoglobin (g/L)	96.6 ± 16.8	99.0 ± 17.1	0.594
Albumin (g/L)	37.1 ± 3.30	37.4 ± 3.20	0.689
Prealbumin (mg/L)	329 ± 70.0	336 ± 54.9	0.706
Triglyceride (mmol/L)	2.20 ± 1.70	1.50 ± 0.60	0.125
Total cholesterol (mmol/L)	4.60 ± 1.10	4.00 ± 0.80	0.034
hsCRP (mg/L)	3.20 (1.60–8.60)	2.30 (1.10–9.40)	0.486
IL-6 (pg/mL)	8.80 (5.80–17.1)	8.80 (6.30–12.6)	0.888
IL-1*β*, (pg/mL)	5.00 (5.00–7.40)	5.00 (5.00–9.50)	0.666
TNF-*α*, (pg/mL)	14.2 (12.4–16.7)	12.4 (11.4–15.0)	0.161
TGF-*β*, (pg/mL)	157 (1.00–434)	276 (142–677)	0.221
HGS, (kg)	20.3 ± 9.80	19.8 ± 7.10	0.860
Low-HGS, [n (%)]	75 (79.8)	13 (81.3)	1.000
MAMC, (cm)	21.9 ± 3.50	20.5 ± 2.30	0.117
Lean mass, (kg)	45.8 ± 10.4	46.2 ± 7.80	0.908
Fat mass, (kg)	15.9 ± 6.50	14.1 ± 4.10	0.304
ASMI, (kg/m^2^)	6.80 ± 1.20	6.80 ± 0.80	0.797
Sarcopenia [n (%)]	32 (34.0)	7 (43.8)	0.453

MCS, Mental Component Summary; BMI, body mass index; BUN, blood urea nitrogen; Kt/V, dialysis efficiency index; hsCRP, high-sensitivity C-reactive protein; IL-6, interleukin-6; IL-1β, interleukin-1β; TNF-α, tumor necrosis factor-α; TGF-β, transforming growth factor-β; HGS, hand grip strength; MAMC, mid-arm muscle circumference; ASMI, appendicular skeletal muscle index. Continuous variables are expressed as mean ± standard deviation or median with interquartile range in case of nonnormally distributed data, and categorical variables are expressed as percentages.

† Patients were divided into the 2 groups by whether MCS ≥ 50.

‡ Obtained from two-sample t-test, Mann–Whitney U test, or chi-square test as appropriate.

§ Other: IgA nephropathy, membranous nephropathy, drug-induced nephropath.

### Characteristics of study participants

**Figure 1:**
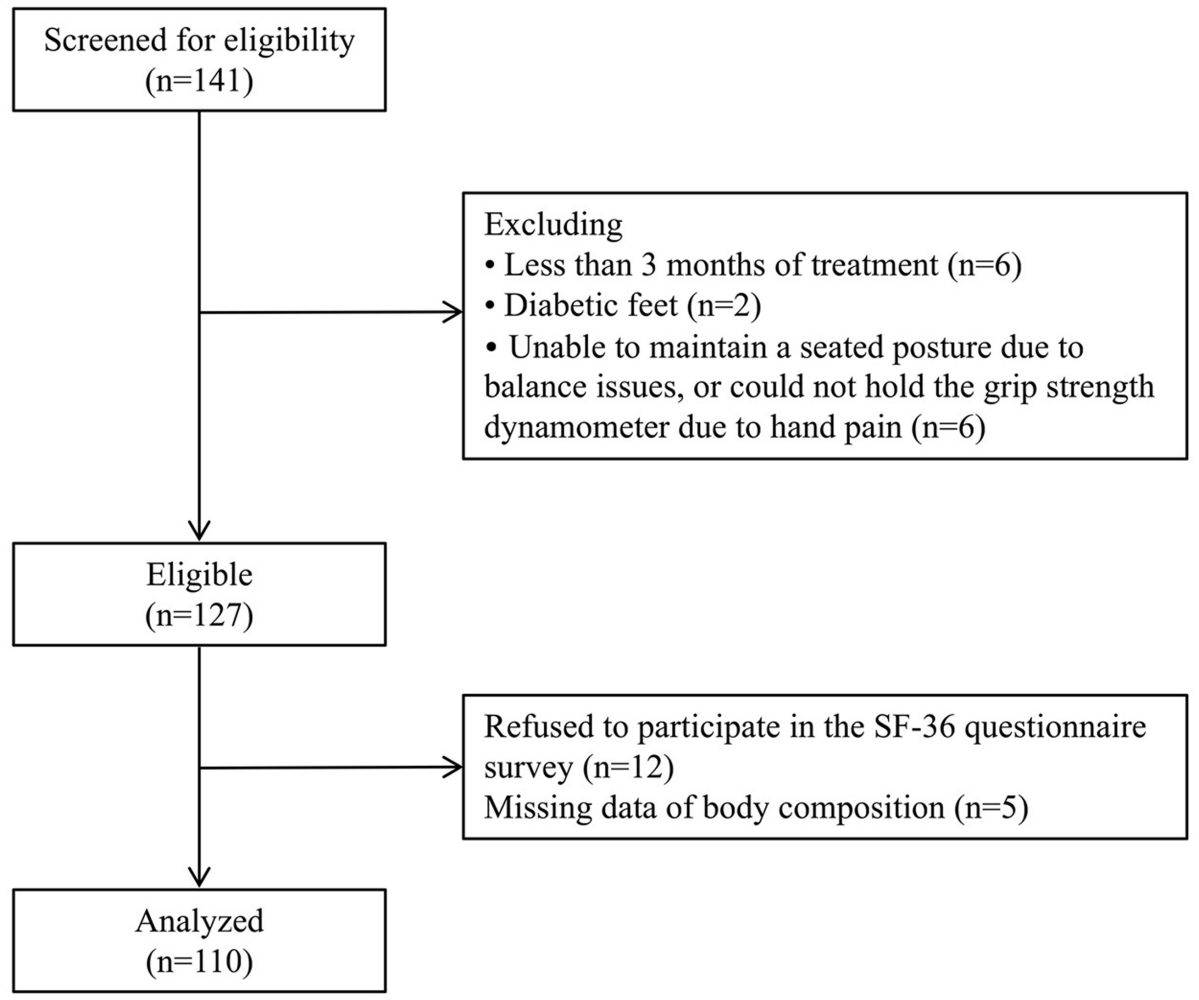
Flow chart for sample selection.

The screening process is presented in Figure 1. Demographic, biochemical and anthropometric parameters of the study participants are shown in Table 3. A total of 110 patients (70 men and 40 women) undergoing MHD were enrolled in the study, with a mean age of 63.9 ± 13.0 years and a median dialysis vintage of 25.5 months (12.0–52.3 months), and 56 of them had diabetes (50.9%). The average BMI was 23.0 ± 3.60 kg/m^2^, the mean HGS was 23.7 ± 9.60 kg in men and 14.3 ± 5.30 kg in women. According to the AWGS criteria, 80% of the participants were diagnosed as low-HGS, 35.5% have low-ASMI, and 35.5% were suffering from sarcopenia. The mean PCS score was 43.8 ± 9.50, and the mean MCS score was 57.3 ± 8.80.

**Table 3:** Demographic, biochemical and anthropometric parameters of the study participants (*n* = 110).

Variables	Mean ± SD, Median [IQR] or n (%)^†^
Age (years)	63.9 ± 13.0
Women [n (%)]	40 (36.4)
BMI (kg/m^2^)	23.0 ± 3.60
Dialysis vintage (months)	25.5 (12.0–52.3)
Cause of end-stage renal disease [n (%)]	
Diabetic nephropathy	39 (35.5)
Hypertensive nephropathy	14 (12.7)
Polycystic kidney disease	4 (3.60)
Unknown causes	42 (38.2)
Other^‡^	11 (10.0)
Diabetes mellitus [n (%)]	56 (50.9)
BUN (mmol/L)	27.5 (23.7–32.0)
Creatinine (μmol/L)	989 ± 294
Kt/V	1.30 (1.20–1.50)
Serum phosphorus (mmol/L)	2.20 (1.80–2.60)
Hemoglobin (g/L)	96.9 ± 16.8
Albumin (g/L)	37.1 ± 3.20
Prealbumin (mg/L)	330 ± 67.7
Triglyceride (mmol/L)	2.10 ± 1.60
Total cholesterol (mmol/L)	4.50 ± 1.10
hsCRP (mg/L)	3.00 (1.50–8.60)
IL-6 (pg/mL)	8.80 (5.90–15.6)
IL-1*β* (pg/mL)	5.00 (5.00–7.40)
TNF-*α* (pg/mL)	14.2 (12.3–16.6)
TGF-*β* (pg/mL)	178 (17.8–530)
HGS (kg)	19.3 ± 9.50
Men	23.7 ± 9.60
Women	14.3 ± 5.30
Low-HGS [n (%)]	88 (80.0)
MAMC (cm)	21.7 ± 3.40
Lean mass (kg)	45.9 ± 10.0
Fat mass (kg)	15.6 ± 6.20
ASMI (kg/m^2^)	
Men	7.20 ± 0.90
Women	6.20 ± 1.20
Low-ASMI [n (%)]	39 (35.5)
Sarcopenia [n (%)]	39 (35.5)
SF-36	628 (577–689)
PCS	43.8 ± 9.50
MCS	57.3 ± 8.80

n, number of participants; SD, standard deviation; IQR, interquartile range; %, percentage of participants; BMI, body mass index; BUN, blood urea nitrogen; Kt/V, dialysis efficiency index; hsCRP, high-sensitivity C-reactive protein; IL-6, interleukin-6; IL-1β, interleukin-1β; TNF-α, tumor necrosis factor-α; TGF-β, transforming growth factor-β; HGS, handgrip strength; MAMC, mid-arm muscle circumference; ASMI, appendicular skeletal muscle index; SF-36, Study Short Form 36; PCS, Physical Component Summary; MCS, Mental Component Summary.

† Continuous variables are expressed as mean ± standard deviation or median with interquartile range in case of nonnormally distributed data, and categorical variables are expressed as percentages.

‡ Other: IgA nephropathy, membranous nephropathy, drug-induced nephropathy.

### Associations between HGS and PCS in patients undergoing MHD

After adjustment for age, sex, fat mass, ASMI, creatinine, TC, hsCRP, and albumin, the five-model multivariate binary logistic regression analyses showed that lower HGS levels and HGS <28 kg for men and < 18 kg for women were both independently associated with lower PCS scores in patients undergoing MHD (*p* < 0.05; Table 4). Since the univariate analysis of HGS and MCS scores did not show statistical significance (Table 2), subsequent binary logistics regression analysis was not conducted in MCS.

**Table 4:** Results of multivariate binary logistic regression analyses of low-PCS (dependent variable) according to related HGS indicators (independent variables).

	Model1^†^	Model2^‡^	Model3^§^	Model4^¶^	Model5^††^
HGS (kg) as a continuous variable					
Odds ratio	0.917	0.917	0.917	0.917	0.917
95%CI	0.871–0.966	0.871–0.966	0.871–0.966	0.871–0.966	0.871–0.966
*p* value	0.001	0.001	0.001	0.001	0.001
HGS (normal/Low HGS) as a categorical variable					
Normal-HGS	Ref	Ref	Ref	Ref	Ref
Low-HGS					
Odds ratio	5.25	5.25	5.36	5.36	5.36
95%CI	1.94–14.2	1.94–14.2	1.89–15.2	1.89–15.2	1.89–15.2
*p* value	0.001	0.001	0.002	0.002	0.002

PCS, Physical Component Summary; HGS, handgrip strength; CI, confidence interval; TC, total cholesterol; hsCRP, high-sensitivity C-reactive protein.

† Model 1: Unadjusted.

‡ Model 2: Adjusted for age and sex.

§ Model 3: Adjusted for model2 plus fat mass and ASMI.

¶ Model 4: Adjusted for model3 plus creatinine, TC and hsCRP.

†† Model 5: Adjusted for model4 plus albumin.

## Discussion

To our knowledge, this study is the first to investigate the relationship between HRQoL and sarcopenic parameters, with a specific emphasis on muscle strength, within the Chinese hemodialysis patient. Our findings suggest that HGS was independently associated with a better quality of life, predominantly reflected in the PCS score of the SF-36. However, no significant correlation was found in the MCS domain.

In our study, the mean SF-36 PCS was 43.8 and the mean SF-36 MCS was 57.3, which closely aligns with previous study’s findings of PCS 46.3 and MCS 55.1 in Romania (30). However, our study exhibits substantial discrepancies when compared to research conducted in Russia (31). Studies from different countries have shown that there are differences in HRQoL among ESRD patients treated with hemodialysis. A study involving MHD patients from three continents demonstrated significant variations in HRQoL across various countries and regions (11). Patients in the United States had the highest MCS scores, and Japanese patients reported better physical functioning than those in the United States or Europe. PCS scores across various studies ranged from 33.1 to 46.3, while MCS scores ranged from 43.2 to 55.1 (11, 30, 31). The possible reasons for the existence of these differences are potentially due to variations in sample size, demographic characteristics, comorbidities, social support, and dialysis duration.

In the present study, the low PCS group was associated with lower values for parameters related to muscle strength, such as HGS, older age, and lower creatinine levels, as well as with higher levels of inflammation, such as hsCRP and IL-6, and higher levels of TC and body fat mass. The low-PCS group exhibited a significantly higher mean age compared to the high-PCS group. It may be due to a decrease in physical activity levels with aging, and a reduction in social activities as well. Fat mass in the high-PCS groups was lower than the low-PCS group. One possible explanation is that individuals in the high-PCS group tend to participate in more social activities and exhibit a higher level of physical activity. Increasing physical activity levels has been shown to be advantageous for promoting an increase in lean body mass and a decrease in body fat mass. Compared to the high-PCS group, the low-PCS group had higher inflammatory markers (IL-6 and hsCRP). This phenomenon may be mediated by the change in body fat composition. As mentioned above, MHD patients in the low-PCS group had higher fat mass, which is associated with a redistribution of fat depots, shifting from subcutaneous locations to more harmful ectopic locations (32–35). This redistribution results in obesity, particularly visceral fat deposits, and lipid spillover into muscle (36–38). This fat deposition shifts fat into infiltrating skeletal muscle as intermuscular adipose tissue, which releases a host of proinflammatory cytokines, such as interleukin-6 (IL-6), onto muscle fibers, resulting in local inflammation within the muscle (39). Our findings indicated that body fat mass, hsCRP and IL-6 may also serve as one of the indicators for assessing HRQoL in hemodialysis patients. In addition, the results showed that the high-PCS group had lower TC levels. Possible explanations include the fact that, for people in the high-PCS group, they tend to be more active and socially inclined, which may contribute to keep better body composition and blood lipid levels. The research by Lonardo et al. suggested that changes in skeletal muscle mass could have a role in worsening lipid profile (40).

As for the MCS groups, the high-MCS group exhibited higher TC levels. The differences in TC levels between the groups remain unclear. However, one study conducted in Chinese centenarians found that lipid profile was positively associated with the HRQoL (41). These findings warrant further investigation to elucidate the relationship between MCS and TC levels in MHD patient.

In this study, the mean HGS measurement was 19.3 ± 9.50 kg, consistent with other studies on dialysis patients (42, 43). The multivariate logistic regression model revealed that only HGS was significantly associated with the SF-36 PCS scores, indicating that muscle strength determined by handgrip, rather than body composition (fat mass and ASMI), could be an independent factor that lowers physical health of the HRQoL in MHD patients. In previous studies on hemodialysis patients, Maria et al. found that there was no association between sarcopenia and HRQoL, but the components of sarcopenia (handgrip strength and gait speed) showed a correlation with the physical domain of HRQoL (22). Giglio et al. demonstrated that the group with low muscle strength had lower scores in the SF-36 domains, a finding not observed in the group of low muscle mass (23). Similarly, Tsekoura et al. suggested that HGS was significantly correlated with quality of life in hemodialysis patients (42). The associations between muscle strength and HRQoL have also been reported in other populations, such as healthy adults, patients with osteoporosis, cancer survivors, systemic lupus erythematosus patients and spinal disorders patients, suggesting that muscle strength rather than skeletal muscle mass might affect HRQoL (44–48). The mechanism underlying the positive association between muscle strength and HRQoL reported in the aforementioned studies is hypothesized to be mediated by physical activity. Physical inactivity in MHD patients can cause muscle weakness, declines in physical functioning and reduction of atrophic fiber number. The loss of motor neurons leads to an increase in the size of remaining motor units, concurrent with a greater preservation of type I muscle fibers. This results in the preservation of muscle mass with a relatively smaller proportion of type II fibers, thereby contributing to reduced muscle strength (49). Chen et al. conducted a randomized controlled trial in which MHD patients underwent intradialytic low-intensity strength training or stretching exercises (control group) (50). The results showed that compared with the control group, the strength training group showed significant improvements in knee extensor strength and HRQoL domains such as leisure time, physical activity, and self-reported physical function and disability in activities of daily living. Silva et al. also found that supervised physical therapy during hemodialysis sessions improved knee extensor muscle strength, leading to benefits in activities of daily living and HRQoL (51). The current findings suggest that even at lower training intensities, most MHD patients increase muscle strength, minimizing the negative impact of a sedentary lifestyle (23, 51, 52). Maintaining or promoting muscle strength through exercise is crucial for the HRQoL of patients undergoing MHD.

Our results indicated that high HGS was independently correlated with a better quality of life, primarily in the PCS domain. However, no significant correlation was found in the MCS domain. These results align with some prior studies conducted on older populations, (53, 54) but only partially agree with other investigations. In MHD patients and community-dwelling nonagenarians, several studies have substantiated the correlation between HGS and both physical and mental health (23). Sayer et al. reported a correlation between reduced grip strength and a higher prevalence of poor SF-36 score in general health, physical activity and mental health among younger individuals (20). In contrast, Wang et al.’s study did not demonstrate a correlation between muscle strength and PCS or MCS domain scores in MHD patients (55). The discrepancies observed in these studies could be attributed to several factors: (a) Variations in muscle strength measurement methods, such as focusing on ankle dorsiflexion strength (reflecting lower limb muscle strength) or grip strength (primarily reflecting upper limb muscle strength); (b) Inconsistencies in the scales used to measure HRQoL, including the SF-36, EuroQol-5 Dimensions, and disease-specific scales; (c) Diversity in study populations, encompassing age, race, comorbidities, and other demographic factors, which may contribute to differing outcomes. Therefore, further research is necessary to explore the relationship between upper and lower limb muscle strength and HRQoL in hemodialysis patients, with a specific focus on prospective and interventional studies.

In summary, this study found that HGS, body fat mass, IL-6, hsCRP and TC were associated with HRQoL in a univariate analysis. Multivariate logistic regression model suggested that HGS may serve as a robust predictor of HRQoL in patients undergoing MHD. However, further research is warranted to validate these findings. The major strength of our study is that it firstly analyzed the relationship between HGS and HRQoL in Chinese patients undergoing MHD. Moreover, prior research has suggested that multiple factors may impact HRQoL scores and muscle health in MHD patients (43, 56–58). We considered potential confounders—age, sex, fat mass, ASMI, creatinine and TC levels, as well as inflammatory markers such as hsCRP and nutritional indicators such as albumin—thus bolstering the reliability of our results. The study faced several limitations. Firstly, due to its cross-sectional design, the study cannot establish any cause-effect relationships between muscle strength and HRQoL. Secondly, the analysis excluded certain sociodemographic variables such as education level, marital status, and living arrangements, as well as factors like depression and sleep quality. These omissions may have affected the potential for robust correlations between muscle strength and HRQoL, including dietary intake and nutritional status, which are also known to affect the quality of life of MHD patients (18, 56, 59). Furthermore, the participants of this study were selected from a single hemodialysis center using a convenience sampling approach. Future research aims to enhance the robustness of the findings by increasing the sample size, adopting more rigorous sampling methods, and conducting multi-center studies to explore the relationship between muscle strength and quality of life.

## Conclusion

In conclusion, HGS might represent a simple and reliable screening tool for assessing HRQoL, particularly in relation to physical health, in patients undergoing MHD. The management of muscle strength may play a pivotal role in improving HRQoL in MHD patients.

## Glossary

**Table:** 

HRQoL	Health-related quality of life
MHD	maintenance hemodialysis
SF-36	Short Form 36 Health Survey
PCS	Physical Component Summary
MCS	Mental Component Summary
HGS	handgrip strength
ESRD	end-stage renal disease
PF	physical functioning
RP	role limitations due to physical problems
BP	bodily pain
GH	general health
VT	vitality
SF	social functioning
RE	role limitations due to emotional problems
MH	mental health
AWGS	Asian Working Group for Sarcopenia
BUN	blood urea nitrogen
TC	total cholesterol
hsCRP	high-sensitivity C-reactive protein
IL-6	interleukin-6
IL-1*β*	interleukin-1*β*
TNF-*α*	tumor necrosis factor-*α*
TGF-*β*	transforming growth factor-*β*
BMI	body mass index
TSF	triceps skinfold thickness
MAC	mid-upper arm circumference
MAMC	Mid-arm muscle circumference
ASM	appendicular skeletal muscle
ASMI	appendicular skeletal muscle index

## Data Availability

The raw data supporting the conclusions of this article will be made available by the authors, without undue reservation.

## Footnotes

^1^https://www.chictr.org.cn
